# Evaluating Engagement in a Digital and Dietetic Intervention Promoting Healthy Weight Gain in Pregnancy: Mixed Methods Study

**DOI:** 10.2196/17845

**Published:** 2020-06-26

**Authors:** Jane C Willcox, Daniel Chai, Lawrence J Beilin, Susan L Prescott, Desiree Silva, Cliff Neppe, Rae-Chi Huang

**Affiliations:** 1 La Trobe University Bundoora Australia; 2 Telethon Kids Institute Perth Australia; 3 The University of Western Australia Perth Australia; 4 Joondalup Health Campus Shenton Avenue Perth Australia

**Keywords:** pregnancy, internet-based intervention, patient participation, qualitative research, eHealth, body weight, obesity

## Abstract

**Background:**

Early excess and inadequate gestational weight gain (GWG) have been associated with negative outcomes for mother and child. The use of digital media to deliver pregnancy lifestyle interventions is increasing, but there is little data on participant engagement. The Pregnancy Lifestyle Activity and Nutrition (PLAN) intervention pilot study was an electronic health and dietetic-delivered intervention program promoting healthy GWG in early pregnancy.

**Objective:**

This study aims to explore the interactions of participants with the program and to assess its acceptability.

**Methods:**

This study uses both quantitative and qualitative methods using data from parent randomized controlled trial (ACTRN12617000725369). Quantitative data from 22 participants in the intervention arm who completed the study provided measures of the interactions participants had with the digital components of the program and with dietetic consultations. A descriptive qualitative analysis employed semistructured interviews with 9 participants to elicit views on the acceptability of the intervention and its components.

**Results:**

The electronic delivery of information and recording of weight from 8 to 20 weeks of gestation were universally accepted. Component (face-to-face dietitian, weight tracker, website information delivery, and SMS goal prompting) acceptability and engagement differed between individuals. A total of 4 key themes emerged from the qualitative analysis: supporting lifestyle change, component acceptability and value, delivery platforms, and engagement barriers.

**Conclusions:**

The PLAN intervention and its delivery via a blend of personal dietetic consultations and digital program delivery was found to be acceptable and valuable to pregnant women. Individuals responded differently to various components, emphasizing the importance of including women in the development of lifestyle interventions and allowing participants to choose and tailor programs. Larger randomized controlled trials using these insights in a broader section of the community are needed to inform the iterative development of practical, time-efficient, and cost-effective ways of supporting optimal GWG with the potential to optimize outcomes for pregnant women and their child.

## Introduction

### Background

Gestational weight gain (GWG) has emerged as an important health variable contributing to the health of both mother and child. Weight gained outside the recommended Institute of Medicine guidelines [1] across all prepregnancy BMIs is associated with negative short- and long-term health outcomes [2,3]. For example, excess GWG increases the risk of antenatal hypertensive disorders, gestational diabetes, and atypical delivery outcomes [2,4-8], along with maternal, infant, and later life overweight or obesity [4,6]. Over the last two decades, women have increasingly failed to meet GWG recommendations, with more recently an estimated 30% to 65% of women in developed countries, across all prepregnancy BMI categories, exceeding the recommended guidelines for GWG [9,10]. These figures highlight the need for better education directed toward prevention.

Research interest in GWG has seen an increasing number of studies promoting healthy GWG through diet only, exercise only, and combined diet and exercise interventions, compared with standard care [11]. A limitation of many of these interventions is the reliance on support from research staff or health professionals, restricting the reach and scalability to larger sections of the community [12]. More recently, a small number of pilot studies have investigated the digital delivery of GWG interventions using internet and mobile phones (electronic health [eHealth] and mobile health [mHealth]), and the results from these studies show promise [13-15]. Digital delivery potentially offers an opportunity to augment health professional care and provide trusted source information across demographic groups through a low-cost, easy access method [16]. Although a 2017 meta-analysis of the effectiveness of eHealth technologies on GWG found a nonsignificant pooled result in only six studies, it emphasized that the engagement of participants was critical to the success of digitally delivered interventions [13].

Understanding how participants engage and the experiences they have with interventions is crucial to assist in their evaluation and to inform improvement [17,18]. According to O’Brien et al [19], *engagement* may be defined as “the ability to engage and sustain engagement in digital environments.” A number of papers have emphasized the importance of understanding and incorporating the views and engagement metrics of stakeholders into the evaluation of digital interventions [17,18]. For example, in a review of mobile technology in psychosocial and health behavior treatments by Heron and Smyth [17], the authors suggest that interventions need to be more sensitive to individual characteristics and needs of stakeholders. They argued that incorporating end-user feedback into evaluation will facilitate the feasibility and acceptability of interventions. There is a scarcity of literature evaluating participants' engagement with pregnancy and GWG interventions.

The Pregnancy Lifestyle Activity and Nutrition (PLAN) randomized controlled trial aimed to promote healthy GWG in early pregnancy via an eHealth and dietetic-delivered intervention [20]. Women were recruited between 8 and 11 weeks of gestation and randomized to the intervention or routine antenatal care. The 12-week intervention involved one dietetic one-on-one consultation, examining diet and physical activity, and a web-based and SMS program providing diet, physical activity, and well-being advice. The digital component of the program included tracking of GWG, lifestyle goal setting and feedback and diet, physical activity, and mental health information delivered weekly. Participants were then followed up to 3 months postpartum.

A total of 57 women, with a mean age of 33.3 (SD 2) years and a mean gestational age of 9.2 (SD 1.2) weeks, were recruited. The mean BMIs of women in the control and intervention groups at enrollment were 25.3 (SD 5.3) kg/m^2^ and 26.0 (SD 1.3) kg/m^2^, respectively. A total of 43/57 (75%) participants (control group: 19 and intervention group: 24) completed the study. Of the 24 participants in the intervention arm, 22 completed the 12-week intervention program. Although the pilot-sized numbers precluded the power to detect differences in GWG, as well as pregnancy and infant outcomes, infants weighed less in the intervention group (6193 g versus 5405 g; *P*=.01) at 3 months. Furthermore, participants in the intervention arm demonstrated a mean decrease in total fat consumption, increase in fruit serves per day, and score ranking perceived importance of dietary change, compared with participants in the control group. Although limited in number, these pilot results and learnings contribute to the literature on dietetic and digitally delivered antenatal interventions.

### Objective

Consistent with the postevaluation phase of the development and evaluation processes for digitally delivered interventions [18], this study aimed to explore women’s engagement and perspectives on the PLAN eHealth and dietetic intervention [20].

## Methods

This mixed methods study used quantitative assessment for the interaction with the PLAN intervention and qualitative assessment to investigate women’s engagement with and acceptance of the PLAN intervention.

The mHealth Development and Evaluation framework [18] and the Process-Evaluation Plan for Assessing Health Programme Implementation [21] informed the development of this study. The Consolidated Reporting Criteria for Qualitative Studies [22] informed the reporting of this study (Multimedia Appendix 1).

Ethics approval was obtained from the human research ethics committees of Joondalup Health Campus (October 17, 2015; ethics number 1525) and St John of God Health Care (May 26, 2016; ethics number 873). The trial was registered with the Australian New Zealand Clinical Trials Registry (ACTRN12617000725369).

### Quantitative Assessment of Intervention Engagement

Intervention interaction was measured post hoc by program-generated data. The data were extracted from participants in the intervention arm who participated in the 12-week intervention (n*=*22).

#### Data Collection

The characteristics of the participants, including sociodemographic and GWG, were collected from the program data. Indicators of interaction were extracted from the program data files and included retention (numbers completing the trial), dose delivered (contacts delivered), and intervention engagement (contact with the program elements).

The contacts delivered included dietetic consultation, emails with links to lifestyle information and weight tracking prompting, and SMS for goal prompting. Contacts with the program elements included attendance at the dietetic consultation, accessing the web-based app, input into weight tracking, and replies to the goal accomplishments. Data on the frequency and depth of website access were not available.

#### Data Analysis

Quantitative interaction and participants’ characteristics were analyzed with categorical variables reported as numbers and percentages and continuous variables reported as means and SDs.

### Qualitative Assessment of Intervention Engagement and Acceptance

A qualitative descriptive research methodology [23,24] using semistructured interviews was employed to obtain in-depth data from consenting women.

#### Study Participants

Women who had completed the active 12-week phase of the PLAN intervention (n=22) were invited to participate in the qualitative review by the PLAN research coordinator.

#### Data Collection

Telephone interviews were conducted by an independent female interviewer trained in qualitative research methods and not involved in the PLAN study (JCW, a research fellow). The participants were informed that JCW was an independent researcher from a different university and had experience in this qualitative area of research. The content of the standard interview guide (Multimedia Appendix 2) was developed by R-CH. The semistructured and structured questions aimed to elicit women’s views on the PLAN intervention, its components, and potential improvements. In addition, women were asked to rate the experience of the PLAN intervention on helping follow a healthier lifestyle during pregnancy (1=not good and 10=very good). The interviews were digitally recorded with the consent of the participants. Field notes were collected by JCW during and after the telephone interviews.

#### Data Analysis

Data immersion, coding, category creation, and thematic analysis were used to find repeated patterns of meaning across datasets [25,26]. The researchers (JCW and R-CH) used an inductive approach using raw data to derive themes through interpretations made from the raw data [27].

## Results

### Participants

The sociodemographic and GWG characteristics of the women in the total PLAN study are reported elsewhere [20]. The sociodemographic and GWG characteristics of the 22 participants in this substudy are provided in Table 1. The mean age of participants was 33.0 (SD 4.1) years, with a mean gestational age of 9.0 (SD 1.2) weeks at recruitment. The mean self-reported prepregnancy BMI was 25.4 (SD 4.7) kg/m^2^, and one-fifth of the participants (21%) gained weight within the GWG guidelines. The majority of the participants (91%) had private antenatal care, and nearly two-thirds (64%) of those reported that their education held postsecondary qualifications.

**Table 1:** Characteristics of participants in the active Pregnancy Lifestyle Activity and Nutrition intervention arm and qualitative interviews.

Participant characteristic		Participants in the intervention arm who completed the study (n=22)		Qualitative interviews (n=9)	
Maternal age (years), mean (SD)		33.0 (4.1)		33.0 (3.2)	
Enrollment gestational age (weeks), mean (SD)		9.0 (1.2)		9.0 (1.3)	
Reported prepregnancy BMI (kg/m^2^), mean (SD)		25.4 (4.7)		26.7 (4.3)	
Measured enrollment BMI (kg/m^2^), mean (SD)		26.2 (4.9)		28.3 (4.5)	
**Enrollment BMI category (kg/m^2^), n (%)**					
	Underweight (<18.5)		0 (0)		0 (0)
	Normal weight (18.5-24.9)		10 (45)		3 (33)
	Overweight (25.0-29.9)		8 (36)		3 (33)
	Obese (≥30.0)		4 (18)		3 (33)
**Ethnicity, n (%)**					
	White		22 (100)		9 (100)
Private health care, n (%)		20 (91)		9 (100)	
**Household income, n (%)**					
	<AUD $100,000 (US $64,368)		2 (9)		0 (0)
	AUD $100,001-AUD $150,000 (US $64,368-US $96,552)		6 (27)		1 (11)
	>AUD $150,000 (US $96,552)		12 (54)		5 (56)
	Did not answer		2 (9)		3 (33)
**Highest level of education, n (%)**					
	Tertiary		12 (54)		5 (56)
	Other (diploma or trade)		2 (9)		0 (0)
	Secondary or below		5 (23)		1 (11)
	Unknown		3 (14)		3 (33)
**Parity, n (%)**					
	Nulliparous		9 (41)		3 (33)
	Multiparous		12 (54)		3 (33)
	Unknown		1 (4)		3 (33)
Total gestational weight gain (kg), mean (SD)		13.1 (3)		14.7 (4)	
**Gestational weight gain within Institute of Medicine guidelines^a^, n (%)**					
	Below		5 (21)		1 (11)
	Within		5 (21)		2 (22)
	Exceed		12 (54)		6 (67)

^a^Intervention period.

### Quantitative Assessment of Intervention Interactions: Intervention Interaction Data

All women accessed the website during the course of the program. Nearly all women (21/22, 96%) attended the dietetic visit and completed the accelerometer tracking (Table 2). Over the 12-week active intervention period, 31 emails were sent to each participant to notify them of a new release of nutrition, physical activity, and GWG information related to their pregnancy stage. In addition, over this period, a mean of 4.46 (SD 6.24) emails were sent to remind participants to input their weight on the website if this had not been completed. The total number of inputs for weight totaled 23.17 (SD 24.21), ranging from 1 to 92. One-fourth of participants (n=5/22, 23%) received emails notifying them of excessive weight gain (3 participants received 1 notification, 1 participant received 2 notifications, and 1 participant received 3 notifications).

**Table 2:** Intervention interaction data (n=22).

Components of the Intervention		Values	Range
**Program component delivery, n (%)**			
	Dietetic visit with personalized nutrition and physical activity assessment and education	21 (96)	N/A^a^
	Accelerometer wear and feedback	21 (96)	N/A
	Website access^b^	22 (100)	N/A
**Email engagement^c^, mean (SD)**			
	Emails sent regarding new material on the web	31 (0)	31-31
	Number of emails received reminding to input weight	4.46 (6.24)	0-23
	Number of emails received warning of excess gestational weight gain	0.33 (0.75)	0-3
	SMS messages sent prompting SMART^d^ goal	8.5 (1.5)^e^	3-9
	Reply to SMS message prompting SMART goal setting	2.96 (2.65)	0-8
	Weight input: active self-monitoring	N/A	N/A
	Number of weight inputs by participants	23.17 (24.21)	1-92

^a^Not applicable.

^b^Website access data only. Frequency of access not available.

^c^Value reflects the mean number of participants (SD).

^d^SMART: specific, measurable, attainable, realistic, and time-based.

^e^A total of 19 participants received all 9 reminders over the course of the active intervention, 3 opted out at weeks 4, 6, and 9, and hence, they received 3, 5, and 8 SMS messages, respectively.

Women were asked to set a specific, measurable, attainable, realistic, and time-based (SMART) goal [28] to assist them with lifestyle behavior change. SMS messages were sent prompting engagement with the SMART goals women set at the program commencement, with an invitation to reply via return SMS. Overall, 3 women opted out of receiving these messages partway through the intervention, receiving between 3 and 8 SMS messages prompting SMART goal engagement. The remaining participants received the full complement of 9 SMS messages prompting SMART goal engagement over the active 12-week period. Over the 12-week period, women replied on average 2.96 (SD 2.65, range 0-8) times to these messages, with 77% (n=17/22) of participants responding at least once with a range of 0 to 8. The SMS replies from the participants indicating if the SMART goal had been attained was positive on 86%, negative on 10%, and neutral on 4% of occasions.

### Qualitative Assessment of the Intervention: Study Participants’ Characteristics

In total, 11 women agreed to participate in the qualitative study (n=11/22, 50%), with 2 participants withdrawing before the interview. Of the 11 women, 9 were consented and interviewed, with 4 women having completed the total study, including the active and follow-up phases (range 38 weeks’ gestation to 16 weeks postpartum). Of these 5 completed the active phase (range 22-28 weeks’ gestation) and were yet to complete their pregnancy.

The mean age of women was 33.0 (SD 3.2) years, and enrollment BMI was 28.3 (SD 4.5) kg/m^2^. One-third of women were of healthy weight, one-third were overweight, and one-third were obese. All women were identified as white, and 43% of the women had completed tertiary education. The interview length ranged from 22 to 36 min.

### Emergent Themes

All participants spoke about their experiences with the PLAN intervention and the acceptability and usefulness of the intervention and its components and offered suggestions for improvement to support them in attaining a healthy GWG and pregnancy lifestyle. Saturation of themes appeared after 7 participants. In all, 4 key areas of focus for the women included the effectiveness of the PLAN intervention to encourage lifestyle change, individual component acceptability, delivery platforms, and barriers to engagement with the PLAN intervention.

#### Pregnancy Lifestyle Activity and Nutrition Intervention Supporting Lifestyle Change

In general, women reported a positive experience with the PLAN intervention. They were asked to rate their experience of the PLAN intervention with helping follow a healthier lifestyle during pregnancy with *one* (the lowest rating) and *ten* (the highest rating). A total of 2 participants rated the program a *ten,* 3 rated it an *eight*, 3 rated it a *seven*, and 1 rated it a *five*. A participant, who rated the program an *8*, qualified her answer by rating the active part of the program an *8* and by rating the nonactive part after 20 weeks a *two*.

When asked if they would recommend the program to a friend, all but 1 woman answered *yes* and 1 answered *maybe*. Again, 1 participant qualified the answer by saying *yes* if the program was fully active and *no* if it only involved data collection.

The most valuable aspect of PLAN cited by many women was the frequent contact and the level of support and encouragement provided. Overall, women conveyed valuing and liking “the extra support in the background”:

It was nice to feel supported and feel like you are on track. I really appreciated the ladies that worked with the study.

Participant 9

I felt cared for and nurtured through this [pregnancy] period.

Participant 7

#### Different Program Components Judged Acceptable and Valuable

Participants discussed the program components as mutually exclusive elements with different participants citing components that they found of value.

##### Dietitian Consultation

The dietetic consultation, which included assessment of food records, was highly valued by the majority of participants. A total of 4 participants expressed positive *surprise* with the extent of information and assistance that they received, particularly around the food groups that they were missing in their diets.

Another 2 participants suffered *morning sickness* and found the dietitian consultation ill-timed for their medical state. They reported finding it difficult to receive information that encouraged exercise and eating well and would have welcomed a later appointment or advice related to *morning sickness*. They expressed a desire to follow up on the recommended dietary changes or have a reassessment once their *morning sickness* had dissipated.

When asked, all women conveyed that they would find it helpful to have another consultation at around 16 to 20 weeks of gestation:

It would be great to get it again at 16-20 weeks. It is good to get the feedback.

Participant 8

##### Weight Tracker

Most women reported using the weight tracker at least once per week. Many women found value in prompting to weigh, recording their weight, and seeing a visual representation of the weight trajectory. A total of 4 women nominated the weight tracker as one of the most valuable aspects because of its help in “seeing how it progressed” and “reinforced the importance of eating well and being active”:

I looked forward to doing it. It was good to be prompted.

Participant 7

It was reassuring as a woman.

Participant 9

All but 2 women stated that they received reminders to weigh (once per week); 4 women described the frequency of weight input and reminder as acceptable, 1 as too infrequent, and 2 as too frequent.

In all, 3 women did not recall receiving feedback or comments from the weight tracker. One woman reported finding positive feedback but further commented that her weight stayed within the recommended quadrant. Moreover, 3 other women conveyed that they were not happy with the negative feedback, which they found “unhelpful.” They desired only constructive feedback and more of a sense of what to alter:

I would have liked more mindful about your lifestyle feedback.

Participant 7

##### Emails, Websites, and Health Information

All but 2 women reported receiving regular notifications for the release of nutrition, physical activity, and GWG information on the website/web-based app related to their pregnancy stage.

A total of 4 women reported reading all the information, 2 reading more than two-thirds, and 3 had not read any information. Many of the women who read the material found that it provided positive information for a lifestyle change and reinforced key health messages. One woman who read the information found the information did not meet her needs by being “too light”:

I liked the simple terms, not too complex. It made you think about your lifestyle.

Participant 6

[It] reinforced the importance of eating well and being active.

Participant 1

Although many participants found the quantity of information appropriate, 1 woman found the quantity of information overwhelming. This was partly because of not being able to exercise for medical reasons and found the information that did not relate to her difficulty:

It got a bit overwhelming. I felt bad that I couldn’t read and do things.

Participant 8

Most of the women who read the information indicated that they liked the information being “fed slowly.” The quantity of nutrition and exercise information each release was deemed “about right.”

All women expressed a desire for the information to continue beyond 20 weeks until pregnancy completion with information tailored to the issues relevant to pregnancy at that stage (eg, reflux). There were 2 women that stated preferences for the information to be provided at the start of the intervention, allowing them to access the correct information when required.

Participants suggested a range of opportunities for improvements to the information, with the most frequently suggested being more practical information about healthy eating, including meal plans and recipes. Additional structured guidance about physical activity was desired by 3 women with more information about the later stages of pregnancy. A few women desired more well-being information:

More information on well-being I think. I was neglectful of myself.

Participant 7

Moreover, 2 women suggested a more up-to-date “look and feel” and cited “Mind the Bump” and “Huggies” websites as having looks and functionality that they liked.

##### Goal Setting

Setting SMART goals was described as being “helpful” by some participants. The goals stopped them from becoming “complacent” and were good “external motivation.” The importance of the timing of text messages was mentioned by a few:

It was good to have a reminder.

Participant 8

The texts were frustrating when not in that space.

Participant 4

Another woman reported that she set a physical activity goal that she had already met for the sake of the program. She conveyed that she was not a “big goal setter.”

Short-term goals that changed regularly were suggested by 4 women, and 2 women would find continuation of the goal setting beyond the active 12-week program.

##### Delivery Platform as a Positive and Familiar Source of Information and Contact

The electronic delivery of information and recording of weight was seen as positive, with women being familiar with this mode of delivery:

We are used to those sort of tools with Fitness Pals and others.

Participant 9

When prompted, women suggested multiple delivery platforms to meet different needs for different times:

Everyone loves an app now.

Participant 9

Texts with links to the information would be good as well as an app and a website.

Participant 7

Some women viewed that the addition of a private Facebook page or community forum would be positive, with its ability to allow interaction between women of different pregnancy stages:

It would be great to build rapport with other ladies going through the pregnancy journey.

Participant 7

Others were concerned about the intrusiveness of Facebook and not wanting to engage in a community where there was a burden of stress:

I have avoided it [Facebook] because of all the worriers on there. It depends on how it was structured.

Participant 9

##### Barriers to Engaging in a Pregnancy Lifestyle Program

Barriers to engaging with the PLAN program were mentioned by all participants in the context of barriers to program engagement and barriers to changing lifestyles.

Program-related difficulties included “glitches,” “freezing,” or “incorrect plotting” being reported by many participants. One participant recounted receiving a message telling her that she had gained excess weight when it did not plot that way on the graph. Another described weight being plotted on the graph in the incorrect section. Several women reported that the information was not accessible on mobile devices, which limited information access at convenient times.

Many women reported surprise in the cessation of the program, including the ability to track weight, beyond the active stage of the PLAN intervention at 20 weeks:

I was not mentally prepared for it [the weight tracker and program] to stop.

Participant 6

All but 1 woman wanted to be able to track weight and have the program continue till pregnancy completion to serve as a reminder to keep them on track with regard to lifestyle:

I would have liked to see it all the way through to after the baby was here. The numbers don’t lie.

Participant 7

Women frequently cited barriers to maintaining a healthy lifestyle and following the guidelines set by the PLAN intervention. Morning sickness was a key reason given by 4 women, reflecting the early gestation commencement of the PLAN intervention. Work, family, and time constraints were the next most commonly discussed constraints:

I didn’t use it all cause of working 52 hours a week.

Participant 3

Back pain and other pregnancy-related physical ailments were also cited, particularly as barriers to physical activity:

I had more pain in my second pregnancy.

Participant 9

A common barrier and concern expressed was the perceived inability to access ”pregnancy-safe” food when eating out. Therefore, some women concluded that it felt easier and safer to purchase unhealthy food that was more likely to be food safe and listeria free.

## Discussion

This paper investigated both the quantitative and qualitative engagement of women in the PLAN intervention. The data contribute to the literature on the engagement and acceptability of digitally supported interventions in pregnancy. Of the women who completed the program, the majority accessed the dietetic consultation and the website. Most women read the health information provided and interacted and tracked GWG, whereas fewer women interacted with or replied to the SMART goal–monitoring SMS messages. A total of 4 key areas of focus were elucidated from the qualitative assessment, including the effectiveness of the PLAN intervention to encourage lifestyle change, individual component acceptability, delivery platform acceptability, and barriers to engagement with the PLAN intervention. Overall acceptance of the program was high, with the social support being offered identified as key. The components (dietitian consultation, weight tracker, health information, and goal setting) were identified as mutually exclusive elements of the intervention with variable acceptance. Although there was a consensus that the electronic delivery of information and recording of weight was positive and universally accepted, the cited barriers to adherence varied among the women. Suggestions for improvement of trial design, including an extension of the active program throughout pregnancy, will be incorporated into future program iterations.

The perceived support provided by the intervention program was a strong theme elicited from the women. This supports the findings of others who have found that health interventions, including digital support, can engender a continued sense of support and community [29-31]. Indeed, social support is an important technique [32] in lifestyle pregnancy interventions that have successfully altered GWG [15,21,33]. Digitally delivered interventions provide the opportunity for more frequent interactions delivered beyond the traditionally delivered environments [31].

This intervention mapped many of the key behavioral change variables required for behavior change [34], including providing information on the behavior-health link, goal setting, review of goals, and self-monitoring. The inclusion of behavior change techniques may be most effective when combined with dietary advice in GWG interventions [35]. Digital technologies capitalize on the ability to extend the reach of intervention delivery beyond a health professional appointment and expand the range of behavior change techniques that can be included [36]. Evidence for blended health professional and digitally delivered interventions is still limited to a small number of studies, and further research is needed to explore what types of blended interventions may be most effective and acceptable to both women and health professionals.

Individual participants identified greater affinity for different intervention components. Although some women identified the weight tracker as one of the most valuable components, others nominated the health information or dietitian visit as key. This highlights the importance of offering a suite of products and interactions that allows women to tailor their own programs and interactions according to their preferences, including personal physical and mental health and social support. This is consistent with a study by Willcox et al [37], where both health professionals and women articulated that the individual requirements of women would be best served by interventions that integrated multiple technology elements. This was seen to serve the individual acceptability and use of different technologies and also the needs of different learning styles. Women saw their ability to access different technologies and elements as a way to self-manage or control information acquisition that was unavailable in traditional care models and information sources. Heron and Smyth argue in their review of mobile technology in psychosocial and health behavior research that interventions need to be more sensitive to the individual characteristics and needs of stakeholders [17]. Further investigation with larger trials will be required to understand the underlying differences in characteristics that underpin this and understand how researchers may segment users and target them most appropriately.

One important finding of this study is that the recording of and education around weight was universally accepted by women. The weight tracker was consistently used by many women, and the qualitative interviews elicited positive views on the practice and prompting of weighing. Self-monitoring is known to be a powerful behavior change technique and is frequently adopted in technology interventions [38]. Feedback from all but 1 woman demonstrated the desire to be able to track weight though to pregnancy completion. Previous research has highlighted the need for women to understand their targeted GWG and allow tracking [39]. Although GWG is well understood to impact the health outcomes of both mother and child, concerns are often raised by health professionals as to the appropriateness of weight discussions with pregnant women [40,41]. Health professionals often cite concern for the physical and psychological health of pregnant women and worries about the perceived negative impacts of weight discussions. This study adds to the data supporting that the majority of women are accepting of GWG discussions and tracking. Further research could engage a wide range of health professionals in supporting digital health augmentation to their counseling to ensure that GWG messages are consistent and professionals are equipped with the necessary skills to address weight management in pregnancy.

### Strengths and Limitations

A strength of this study was the use of a mixed methods design incorporating both quantitative engagement data and qualitative engagement and acceptability data. The pilot size of the original PLAN study, and hence the qualitative work, may be viewed as a limitation. Despite this, the application of qualitative research methods was able to define certain themes. Furthermore, the additional corroborating objective measures of engagement, such as frequency of weight entry and SMS response, correlated well with what was reported in the interviews, providing greater evidence for the integrity of the information obtained. Larger-scale studies with analysis of participant engagement and health outcomes will allow further exploration of engagement variables. Another limitation of the study was the recruitment of predominantly private patients from an upper socioeconomic stratum, with the complexity of recruiting early gestational age participants under public care. Recruiting more broadly and tailoring and testing the intervention on a broader section of the community is required. That only half of the women agreed to participate in the qualitative interviews may be viewed as a limitation. Although saturation was reached after 7 participants, the nonresponders may have held alternate views.

### Conclusions

This study investigated the engagement with and acceptability of a combination of eHealth and dietetic intervention to promote healthy GWG in pregnant women. Women universally found the blended model of a dietitian consult and digital information and tracking acceptable and opportune. However, participants responded more favorably to different elements of the intervention and described a range of factors to enhance future interventions in preventing excess weight gain in pregnancy. This pilot study contributes to the literature on participant engagement and provides participant-related themes, including the importance of choice and tailoring, to inform future research.

## Appendix

Multimedia Appendix 1 The Consolidated Reporting Criteria for Qualitative Studies for this study.

Multimedia Appendix 2 Interview questions (PLAN intervention).

## Abbreviations

eHealth: electronic health
GWG: gestational weight gain
mHealth: mobile health
PLAN: Pregnancy Lifestyle Activity and Nutrition
SMART: specific, measurable, attainable, realistic, and time-based
