# Supplementary material for: Evaluating Engagement in a Digital and Dietetic Intervention Promoting Healthy Weight Gain in Pregnancy: Mixed Methods Study
Source: J Med Internet Res. 2020 Jun 26;22(6):e17845. doi: 10.2196/17845 (PMC7380982; doi:10.2196/17845)
Supplement: Multimedia Appendix 2 [file jmir_v22i6e17845_app2.docx]

**Appendix 2: Interview questions (PLAN intervention)**

1. On a scale of 1-10 (where 1 is ‘not good’ and 10 is ‘really good’) how would you rate your experience in the PLAN trial so far?
2. What aspects of the trial do you most value? (Goal SMS reminders, on line weight tracker, online information, seeing the dietician)
3. Are there any aspects of the trial that you don’t like?
4. Are there any aspects of the trial that you feel could be improved?
5. Do you have any comments (good or bad) regarding the website and weight tracker?
6. What gets in the way of making good lifestyle choices?
7. Do you think that reminders to log in are too frequent, just right,  not frequent enough?
8. Do the email reminders prompt you to read new material?  Would SMS be better?  Does time of the day for receiving reminder affect whether you access the new material
9. Website/App
   1. Is material too dense, not dense enough?  Is it hard to read?  Would you  prefer more dot points?
   2. Do you want more or less information or just right?  If more, what.  If less, what.
   3. Do you want more or less info on diet or just right: If more what, if less what.
   4. Do you want more or less info on exercise or just right: IF more what, if less what.
   5. Do you want more or less info on well-being or just right: If more what, if less what.
   6. Is there anything you would like to have received earlier?
   7. Do you think you would be more likely to read it if you got access to everything at once, instead of gradually?
   8. Does it make any difference to you to access form website or App?  Would making into App make it more likely for you to use?
10. Would you like to continue receiving more information past 20 weeks?
11. Would you like to continue the goal SMS past 20 weeks?
12. Would you like to continue the weight tracking graph past 20 weeks
13. Have you received a negative BMI warning?  How did it make you feel and what would help motivate you?
14. Was the session with the dietician appropriate and helpful?
15. If offered would you like to join a Facebook forum with other women in the trial?
16. If offered would you like or find it helpful to have another session with the dietician?
17. Would you recommend the PLAN program to a friend?
